# Salicylic Acid in Rheumatism

**Published:** 1878-01

**Authors:** 


### Salicylic Acid in Rheumatism.

In the article in the *Lancet*, Dr. Julius Pollock writes:—

It is probable that salicylic acid is the active agent in either case, just as the iodine is the active agent in iodide of potassium; but crude iodine is rarely given now, and in a short time I believe the salicylate of soda will be used in all cases where the action of salicylic acid is desired. It is very soluble, which the acid is not, and it is far less liable to give rise to unpleasant symptoms. I give the preference most decidedly to the soda salt as at present advised, though it is quite possible, indeed likely, that combinations of salicylic acid with potash, ammonia, and iron, may turn out very valuable. In any case of articular rheumatism, whether acute, subacute, or chronic, the salicylate of soda should be tried in doses often, fifteen, or twenty grains, every two, three, or four hours, according to the severity of the symptoms. It is best to give it alone, or combination with a little spirits of chloroform or syrup of orange. As a rule, the good effects of a drug are apparent after eight or ten doses ; the temperature falls rapidly to normal, or even a little below, the pain and swelling of the joints disappear, and the patient is practically convalescent in two or three days ; but it is better to keep up the action of the medicine for a week or so, as relapses are apt to occur if it be discontinued too soon. In some intensely rheumatic subjects it will be necessary to give it again and again before the disease is subdued, and these cases have been used as an argument against its efficacy. Some persons will not admit the value of mercury and iodide of potassium in the treatment of syphilis, and others question the protective power of vaccination against small-pox. All new remedies have to encounter the opposition of ignorance and prejudice, but the evidence in favor of salicylate of soda in the treatment of articular rheumatism is becoming so overwhelming that its great value must shortly be thoroughly established.

No doubt the drug every now and then produces disagreeable symptoms, sickness, deafness, tinnitus aurium, and sometimes a peculiar cerebral disturbance; but these quickly vanish on a discontinuance of the medicine, which may usually be again given in a short time without any such result, In the earlier trials, when the salicylate was not quite pure, these objectionable symptoms were much more common than now. Dr Murchison has suggested, in an able paper read before the Clinical Society on the 25th of May, that the disagreeable effects of the remedy are due to suppression of the function of the kidneys, and he found albumen in the urine of patients who were taking the salicylate of soda, even when the drug was quite pure. This may be so, but at present I have been unable to collect any evidence on the subject.—*Med. and Surgical Reporter.*

